# Primary Aldosteronism and Bone Metabolism: A Systematic Review and Meta-Analysis

**DOI:** 10.3389/fendo.2020.574151

**Published:** 2020-09-25

**Authors:** Shaomin Shi, Chunyan Lu, Haoming Tian, Yan Ren, Tao Chen

**Affiliations:** ^1^Department of Endocrinology and Metabolism, Adrenal Center, West China Hospital of Sichuan University, Chengdu, China; ^2^Department of Endocrinology and Metabolism, Xiangyang Central Hospital, Affiliated Hospital of Hubei University of Arts and Science, Xiangyang, China

**Keywords:** primary aldosteronism, osteoporosis, bone metabolism, fracture, systematic review

## Abstract

**Background:**

Currently, increasing evidence shows that excess aldosterone may have an impact on bone health, and primary aldosteronism (PA) may be a secondary cause of osteoporosis. This problem is worthy of attention because secondary osteoporosis is always potentially reversible, which affects the selection of treatment for PA to some extent. The present systematic review will assess and summarize the available data regarding the relationship between PA and osteoporosis.

**Methods:**

Pubmed and Embase were searched for clinical trials related to the association between PA and bone metabolism. The results were limited to full-text articles published in English, without restrictions for the publication time. The quality of clinical trials was appraised, and the data were extracted. Biochemical parameters of bone turnover in PA patients were assessed using random-effect meta-analysis. Descriptive analysis was performed for other parameters, for data is insufficient.

**Results:**

A final total of 15 articles were included in this review. The meta-analysis of six studies showed that subjects with PA had higher serum PTH levels (*MD*=21.50 pg/ml, 95% CI (15.63, 27.37), *P*<0.00001) and slightly increased urinary calcium levels (*MD* = 1.65 mmol/24 h, 95% CI (1.24, 2.06), *P* < 0.00001) than the EH controls. PA is associated with an increased risk of bone fracture. Bone loss in patients with PA may be reversed by MR antagonists or adrenal surgery.

**Conclusions:**

PA may be a secondary cause of osteoporosis and is associated with an increased risk of bone fracture. The clarification of the relationships between PA and bone metabolism requires additional prospective randomized controlled studies in a large sample.

## Introduction

Primary aldosteronism (PA) is a major cause of secondary hypertension, accounting for approximately 5% to 11% of all cases of hypertension (1–3). Osteoporosis is a disease that requires comprehensive management because many risk factors may affect it. Secondary osteoporosis is always potentially reversible. The possibility of skeletal damage caused by glucocorticoids has been well known for many years (4–7); however, less is known about the link between hyperaldosteronism and osteoporosis. Currently, increasing evidence shows that excess aldosterone may have an impact on bone health, and PA may be a secondary cause of osteoporosis (8, 9). Some studies have indicated that subjects with PA had higher serum parathyroid hormone (PTH) levels, lower serum calcium levels, and higher urinary calcium levels than controls; however, not all studies have supported this finding (10–13). On the other hand, discrepancies also exist in the changes in biochemical parameters after surgery or spironolactone treatment in PA patients (13–15). Moreover, for bone mineral density (BMD) and bone turnover markers, the results of a limited number of studies are controversial. Some researchers have suggested that it is not appropriate to use dual X-ray absorptiometry (DXA) to detect bone damage because PA appear to impair the bone microarchitecture rather than bone density (12, 15–18). Only a few studies have focused on fracture, which is the endpoint event of osteoporosis (15, 17, 19).

Therefore, we conducted this study to systematically review the relationship between primary aldosteronism and bone metabolism according to the available data regarding the biochemical parameters, bone mineral density, bone quality, and risk of fractures of patients with PA. In addition, changes after surgery or spironolactone treatment are reviewed. Finally, the mechanism influencing the effects of excessive aldosterone on bone metabolism will be summarized.

## Methods

### Literature Search

This systematic review was performed according to the Preferred Reporting Items for Systematic Reviews and Meta-Analysis (PRISMA) (20). PubMed and Embase were searched systematically for articles relating to PA and bone metabolism. Searching term was “(primary aldosteronism) AND (osteoporosis OR BMD OR PTH)”. There was no time restriction made on our search. The cutoff time for retrieval was July 2019. All reference lists from the main studies and relevant reviews were screened manually for additional eligible studies. The results were limited to full-text articles published in English.

### Selection Criteria and Quality Assessment

The systematic review question was whether PA was associated with osteoporosis. Two authors independently screened the titles and abstracts for inclusion of all the potential studies identified before. The full-text was retrieved if necessary. All the human studies were included in this evaluation. The literature quality evaluation was conducted by means of the Newcastle-Ottawa Quality Assessment Scale by the two authors independently. NOS included eight items, with total score being nine, and focused on three areas, including participant selection, comparability of study groups, and ascertainment of exposure (21).

During the study selection process for Meta-analysis, inclusion criteria included: participants were patients with PA; PA was confirmed by a saline infusion test or captopril challenge test; the comparison between patients with PA and essential hypertension; studies provided data of serum PTH or calcium levels; case-control study, observational study, cohort study, or randomized clinical trial. Exclusion criteria included: *in vitro* or laboratory study; animal study; review or case report; an abstract or conference; and not meet the inclusion criteria.

### Data Extraction

Data extraction was conducted by the two authors using a standardized data collection form. The following information is obtained from included studies: type of study, country, first author’s name, and year of publication, target population, comparison, all data related to bone metabolism, including biochemical parameters, parameters of bone damage and fractures, and follow-up data after treatment.

### Statistical Analysis

We systematically analyzed all the related parameters of bone metabolism in PA patients both before and after treatment. When parameters were focused only by few studies, descriptive analysis was performed. However, if data is sufficient, Meta-analysis was performed using ReMan5.4. The mean differences (MD) were calculated using random-effects models for the presence of heterogeneity (*I^2^* >50%, P<0.05). *P* value < 0.05 was considered as statistically significant.

## Results

**Figure 1:**
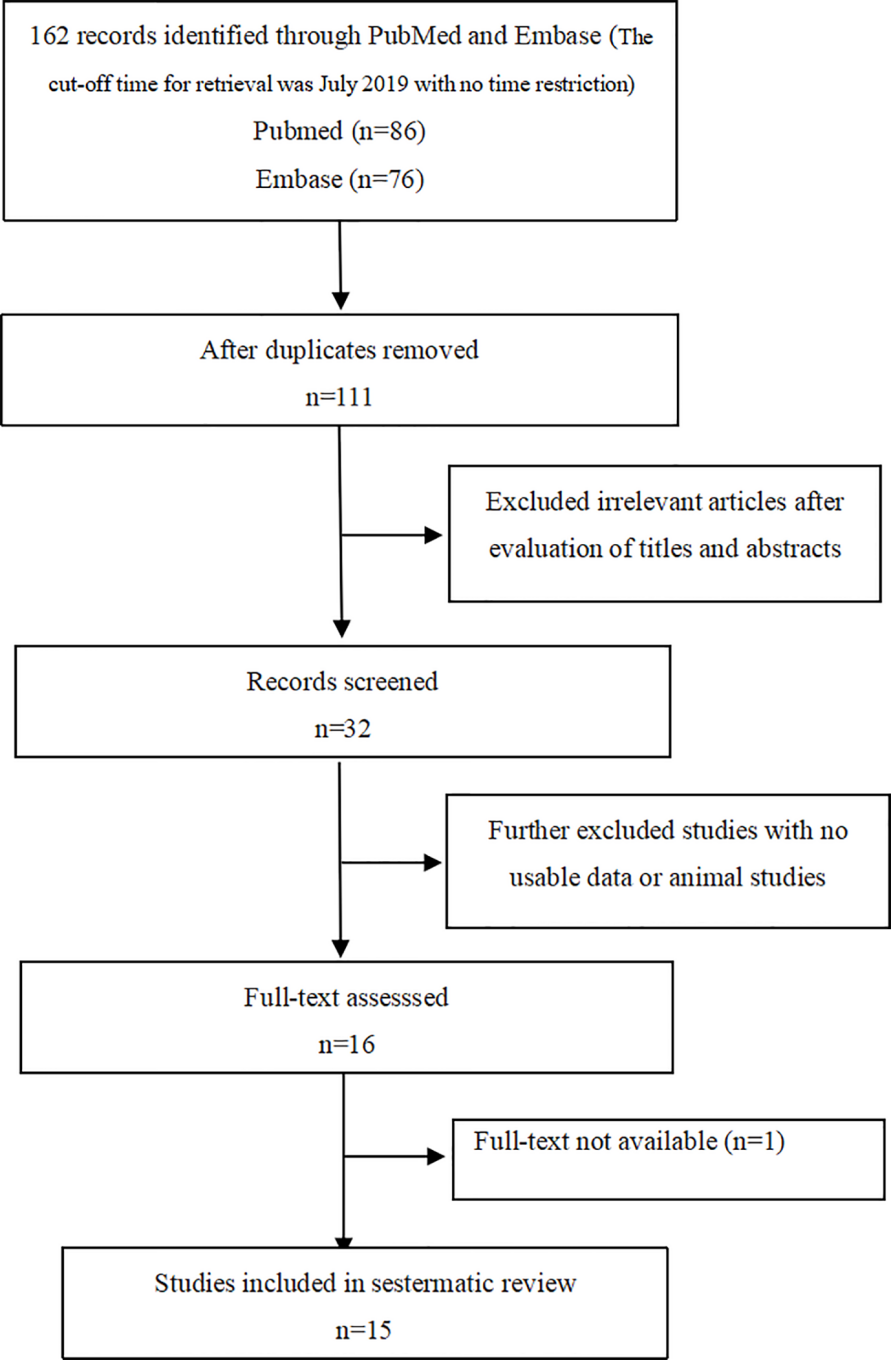
Flow diagram of study selection.

A total of 162 papers were identified, of which, 16 studies were included by screening the titles and abstracts. 1 full-text is not available, and finally, 15 available studies regarding the association between PA and bone metabolism were included in the systematic review (as shown in **Figure 1**) and are summarized in **Table 1**, which included 1 cohort, 1 descriptive, and 13 case-control studies. Participants in these studies came from different regions, including America, Italy, Germany, Japan, Korea, and China. 6 studies were included in the meta-analysis, and the clinical and biochemical parameters of the patients of the 6 studies were shown in **Table 2**.

**Table 1:** The available studies of PA and bone metabolism.

Study ID	Lawrence 1985 (22)	Rossi et al. (11)	Rossi et al. (23)	Rossi et al. (13)	Salcuni et al. (15)	Maniero et al. (24)	Pilz et al. (14)	Ceccoli et al. (16)	Petramala et al. (12)	Wu et al. (25)	Jiang et al. (26)	Kim et al. (18)	Salcuni, 2017 (10)	Notsu et al. (17)	Xiao Yu, 2018 (27)
Country	America	Italy	Italy	Italy	Italy	Italy	Germany	Italy	Italy	Taiwan	China	Korea	Italy	Japan	China
Study Design	Descriptive	Case-control	Case-control	Case-control	Case-control	Case-control	Case-control	Case-control	Case-control	Cohort	Case-control	Case-control	Case-control	Case-control	Case-control
Object	10PA	10PA	6PA	46APA 12BAH	11APA	44PA	10PA	116PA	73PA	2533 PA	242PA	72PA	213OP	56PA	186OP
Control	–	20EH 10HS	16EH	74EH	15AI	61EH	182EH	110EH	73EH 40HS	1:4EH	120EH	335AI	109HS	56HS	96OE 42HS
Results for the PA group	PTH↑; SCa2+ normal	PTH↑; SCa2+↓	PTH↑; UCa2+↑; SCa2+ no difference	PTH↑(APA vs the other two groups)	PTH↑; UCa2+↑; BMD↓; OP (prevalence): 72.5 vs 20%; morphometric VFs: 45.5 vs 13.3%	PTH↑	PTH↑; SCa2+↓	PTH↑; UCa2+↑; SCa2+↓	PTH↑; UCa2+↑; SCa2+↓; 25(OH)-vitamin D↓; prevalence of OP 38.5 vs 28 vs 25%; prevalence of OE 10.5 vs 4 vs 5%	Prevalence of fracture at any site was 14.4‰ vs 8.3‰ for PA and 11.2‰ vs 6.5‰ for APA	PTH↑; UCa2+↑; SCa2+↓	women with PA had a significantly lower lumbar spine TBS, and there was no difference in the BMD	Prevalence of PA (OP vs HS: 5.2% vs 0.9%) (OP plus hypertension plus hypercalciuria vs HS: 26.1% vs 0.9%). Confounding factors were excluded.	Prevalence of VFs: 44.6 vs 23.2%; HbA1c↑; triglycerides↑; urinary calcium-to-creatinine ratio↑; high-density lipoprotein cholesterol↓	PAC and ARR were elevated; a greater number of false-positives was found (24 vs 7 vs 4%); PAC was negatively associated with the lumbar spine BMD
Follow-up results for PA	SCa2+↑ 3 to 6 months postoperatively	PTH↓; SCa2+↑ after 1 month of spironolactone or 2 months after surgery	–	PTH↑; SCa2+↑; UCa2+↓; postoperatively	9/11PA: PTH↓; UCa2+↓; SCa2+↑ (6 months after spironolactone); 5/11PA: BMD↑ (1 year after operation)	PTH↓, SCa2+↑ after operation	PTH↓ 3.7 months after medical or surgical treatment	PTH↓; UCa2+↓; SCa2+↑; BMD↑ 24 months after spironolactone or operation in 40PA	–	–	PTH↓; UCa2+↓; SCa2+↑ after surgery or MR antagonist treatment	–	–	–	–
NOS	–	8	8	7	9	8	9	8	8	8	9	9	8	8	9

PA indicates primary aldosteronism; EH, essential hypertension; HS, healthy subjects; AI, adrenal incidentaloma; APA, aldosterone-producing adenoma; BAH, bilateral adrenal hyperplasia; OP, osteoporosis; OE, osteopenia; UCa2+, urine calcium; SCa2, calcium; BS, trabecular bone score; VFs, vertebral fractures; NTX, type I collagen cross-linked N-telopeptide; BMD, bone mineral density; HbA1c, hemoglobin A1c; VFs, vertebrae fractures; ↑, increase; ↓, decrease; –, no record; NOS, Newcastle-Ottawa Quality Assessment Scale.

**Table 2:** Clinical and biochemical features of the patients of studies included in the meta-analysis.

Study ID	Patient	Number	Median age (year)	Female (%)	BMI (kg/m^2^)	SBP (mm Hg)	DBP (mm Hg)	Aldosterone (ng/dl)	Plasma renin activity (ng/ml/h)	SP (mmol/L)	25-hydroxyvitamin D (ng/ml)
Rossi et al. (11)	PA	10	52.4 ± 12.9	50	23.1 ± 0.8	170.3 ± 17.8*	102.2 ± 3.6*	37.20 ± 11.4*	1 ± 0.02*	3.25 ± 0.64*	–
	EH	10	46 ± 7.2	50	24.1 ± 1.3	166 ± 15.5	104.3 ± 5.9	20.9 ± 9.0	0.87 ± 0.66	3.78 ± 0.35	–
Rossi et al. (23)	PA	16	31–79	–	–	161 ± 3	105 ± 1	29.1 ± 3.1*	0.2 ± 0.04*	–	–
	EH	16	33–69	–	–	157 ± 3	102 ± 1	18.4 ± 4.4	1.14 ± 0.35	–	–
Rossi, 2012 (13)	PA	58	50 ± 12.6	–	–	155 ± 21	94 ± 12	18.7 (11.8–26.7)	0.7 (0.15–1.31)	3.5 ± 0.6	18.6 ± 10.5
	EH	74	50 ± 14	–	–	149 ± 16	93 ± 15	13.5 (9.4–10.4)	1.92 (0.98–6.2)	4.5 ± 0.6	18.9 ± 8.8
Pilz et al. (14)	PA	10	50.1 ± 11	50	31 ± 7.1	179 ± 22	108 ± 12*	33.6 (24.4–67.8)*	PRC 3.1 (2.8–4.4)*	3.2 ± 0.3*	33 ± 23.7
	EH	182	50.2 ± 15.7	59.3	28.5 ± 6	154 ± 23	94 ± 13	16 (12.3–23.4)	11.9 (5.9–28.2)µU/mL	3.9 ± 0.4	30.5 ± 15
Ceccoli et al. (16)	PA	116	51.6 ± 11*	44	27.8 ± 4.8*	158 ± 19*	97 ± 11*	4.98 ± 3.4*	0.4 (0.2–0.7)*	3.7 ± 0.5*	24 ± 15
	EH	110	55 ± 10	68	30.1 ± 5.4	151 ± 15	93 ± 7.5	1.6 ± 1.08	1.6 ± 1.4	4.2 ± 0.3	26 ± 18
Petramala et al. (12)	PA	73	52.5 ± 11.2	–	28.2 ± 4.7*	138.3 ± 16.8*	85.9 ± 11.4*	37 ± 25.1l*	0.9 ± 0.7*	3.8 ± 0.5*	17.8 ± 12.5*
	EH	73	55.6 ± 12.4	–	29 ± 5	131 ± 18.8	82.4 ± 11.2	22.5 ± 13	1.4 ± 1.6	4.2 ± 0.4	32.9 ± 16

In the study of Rossi 1995, EH patients with were divided into two subgroups (LREH, NREH), and the two sets of data were merged. The PA patents in the study of Rossi 2012 were divided into APA group and BAH group, and they were merged too.

PA indicates primary aldosteronism; EH, essential hypertension; LREH, low-renin essential hypertension; NREH, normal-renin essential hypertension; APA, aldosterone-producing adenoma; BAH, bilateral adrenal hyperplasia; BMI, body mass index; SBP, systolic blood pressure; DBP, diastolic blood pressure; PRA, plasma renin activity; PTH parathyroid hormone; PRC, plasma renin concentration; SP, serum potassium; * P<0.05; -, no record.

### Biochemical Parameters, Bone Strength, and Risk of Fractures of Patients With PA

#### Biochemical Parameters of Bone Metabolism

**Figure 2:**
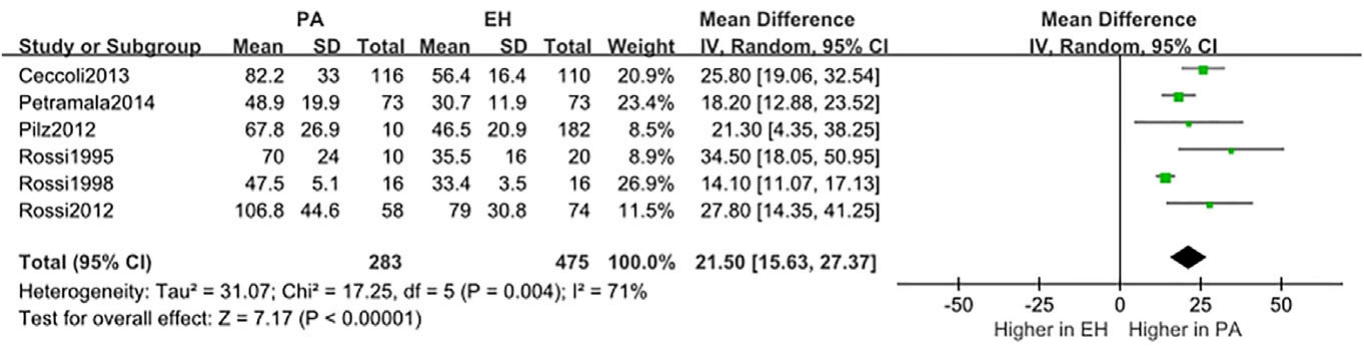
Comparison of PTH in the PA group vs the EH group (pg/ml). Vertical line indicates invalid line; horizontal lines, confidence interval; central squares, statistics; diamond, combined statistic and confidence interval of multiple studies.

**Figure 3:**
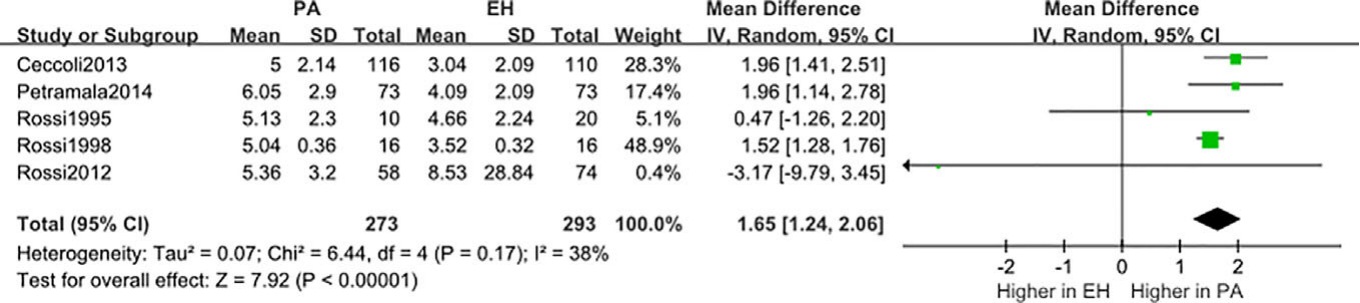
Comparison of urinary calcium in the PA group vs the EH group (mmol/24 h). Vertical line indicates invalid line; Horizontal lines, confidence interval; Central squares, statistics; Diamond, combined statistic and confidence interval of multiple studies.

**Figure 4:**
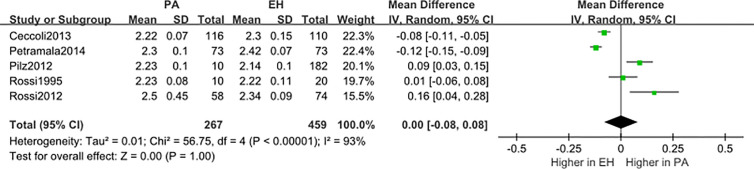
Comparison of blood calcium in the PA group vs the EH group (mmol/L). Vertical line indicates invalid line; horizontal lines, confidence interval; central squares, statistics; diamond, combined statistic and confidence interval of multiple studies.

We conducted a random effect meta-analysis using the available studies to assess the biochemical parameters of bone turnover in PA patients compared with those in essential hypertension (EH) patients. Six studies including 283 PA patients and 475 EH patients were included in the meta-analysis to assess the changes of serum PTH, and the analysis indicated that subjects with PA had higher serum PTH levels than EH patients (*MD*=21.50 pg/ml, 95% CI (15.63, 27.37), *P*<0.00001) (**Figure 2**). Meta-analysis of five studies with urinary calcium levels suggested that urinary calcium was slightly increased in PA patients compared with EH patients (*MD* = 1.65 mmol/24 h, 95% CI (1.24, 2.06), *P*<0.00001) (**Figure 3**), while five studies with serum calcium levels indicated that serum calcium levels were not significantly different between PA and EH patients (*MD* = 0.00 mmol/L, 95% CI (−0.08, 0.08), *P* = 1.0) (**Figure 4**). In the meta-analysis, we only included studies comparing PA patients with EH patients. However, the following three studies compared PA patients with HS (healthy subjects). In 2017, Salicuni compared 12 PA patients with 310 HS and showed that PTH and urinary calcium excretion were significantly higher in PA patients (10), which is in line with the results of previous studies performed by Rossi et al. in 1995 (11) and Petramala et al. in 2014 (12). For the anthropometric parameters and 25(OH)-vitamin D levels, no diﬀerences were found between the PA group and the control group in most studies.

Bone turnover markers (BTMs) have only been mentioned in two studies. Notsu et al. showed that the level of urinary type I collagen cross-linked N-telopeptide was not significantly different in 56 PA patients compared with that in 56 controls (17). Ceccoli et al. showed that the blood level of C-terminal telopeptide of type I collagen I (CTX) tended to be lower and the level of bone-specific alkaline phosphatase (B-ALP) tended to be higher in 40 PA patients after targeted treatment, but these differences were not statistically significant (16).

In summary, PA patients have increased serum PTH levels, and the discrepancies between the studies and the lack of significant changes in serum and urinary calcium levels may be due to insufficient sample sizes and the low impact of aldosterone on bone. Moreover, the laboratory testing methods were inconsistent in different studies, which may affect the accuracy of the conclusion. BTMs did not significantly differ between PA patients and controls, apart from the possible causes of the differences mentioned above. A potential explanation for this is that BTMs reflect only short-term bone transition states rather than chronic cumulative loss.

#### Parameters of Bone Strength

Only a few studies have examined the association of BMD with the prevalence of osteoporosis. Salcuni et al. first reported the association between hyperaldosteronism and osteoporosis in patients with APA. In 11 APA patients, there was decreased BMD in the lumbar spine, total hip, and femoral neck and an increased prevalence of osteoporosis (73% vs 20%) in comparison to those in 15 adrenal incidentaloma (AI) patients (15). Petramala et al. compared 73 PA patients with 73 EH patients and 40 HS and showed that the PA subjects had a significantly higher prevalence of osteopenia/osteoporosis (38.5% and 10.5%) than the EH patients (28% and 4%) and HS (25% and 5%, respectively) (12).

However, there was no difference in the BMD in the lumbar spine and femoral neck between 56 PA patients and 56 HS according to another study (17). Likewise, Kim et al. confirmed that there was no difference in the BMD between 72 PA patients and 335 HS patients (18). Controversial evidence supported the possibility that excess aldosterone may mainly affect bone quality rather than bone mass. Therefore, BMD may not be an ideal index to reflect bone involvement caused by aldosterone. Therefore, the trabecular bone score (TBS), as a parameter representing the bone microarchitecture that is determined by quantifying pixel-level grayscale variations in lumbar spine dual-energy X-ray absorptiometry scans, has been introduced. The TBS is related to the bone microarchitecture and provides information about the skeleton that is not captured by the standard BMD measurement (28). However, TBS itself is also a controversial parameter, which has limited application value in clinical practice. Kim et al. subsequently showed that women with PA had a significantly decreased lumbar spine TBS, and the PAC was inversely correlated with the lumbar spine TBS in women after adjustment for potential confounders. Importantly, all these observations in women remained statistically significant following an additional adjustment for the lumbar spine BMD in the multivariable model. However, similar correlations were not observed in men (18).

In summary, controversial evidence indicated that the BMD evaluated by DXA may not be an early index to reflect the bone involvement caused by excessive aldosterone; therefore, more studies focused on the BMD (e.g., QCT) and bone quality may be needed.

#### Risk of Fractures

Unlike the data on biochemical parameters and BMD, data regarding the risk of fractures are fully concordant and show that PA patients have a higher fracture risk compared with controls. Salcuni et al. first reported that the prevalence of vertebral fractures tended to be higher in PA patients than in controls (45.5% vs 13.3%) (15). Subsequently, Notsu et al. showed that the prevalence of vertebral fractures was significantly higher in 56 PA patients than that in 56 HS patients (44.6% vs 23.2%, P <0.05). Patients with PA suffered severe fractures more frequently than controls (17). However, both of these studies focused on morphometric vertebral fractures rather than fractures with clinical symptoms. Nevertheless, Wu et al. focused on clinical fractures. They used a longitudinal population database from the Taiwan National Health Insurance system that included 2533 PA patients and controls (1:4) and showed that the incidence rate of fracture at any site was 14.4‰ vs 8.3‰ for PA patients and 11.2‰ vs 6.5‰ for APA patients compared with that for controls (*P*<0.05) (19).

In summary, limited evidence has shown that PA is associated with an increased risk of bone fracture, which is of the greatest concern as the end-point event of osteoporosis, but few studies have focused directly on this association, highlighting the necessity of more research.

### Changes in Biochemical Parameters of Bone Metabolism and BMD After Surgery or Spironolactone Treatment

It is well known that secondary osteoporosis is always potentially reversible, so some researchers have examined whether bone involvement can be reversed in PA patients after surgery or spironolactone treatment. Lawrence et al. first observed in 1985 that the serum calcium level was elevated postoperatively in six APA patients (P<0.05) (22). In 1995, Rossi et al. showed an increase in the serum ionized calcium level and a decrease in the PTH level after spironolactone administration or surgical treatment in ten PA patients (P<0.05) without collecting data on calcium excretion (11). In 2012, another study by Rossi et al. confirmed these results in 46 APA patients and found a significant decrease in urinary calcium excretion after adrenalectomy (5.6 ± 3.4 vs 4.1 ± 2.3 mmol/24 h, *P* = 0.038) (13). Subsequently, Maniero et al. also showed that adrenalectomy normalized PTH levels and markedly raised the serum ionized calcium level in 44 APA patients (24). Another study showed that changes in PTH levels appeared to be more pronounced in 5 patients with APA treated by surgery compared with those in 5 patients with idiopathic adrenal hyperplasia (IAH) treated by MR antagonists, but the serum calcium level was not significantly changed. However, this result should be interpreted cautiously since it was obtained in very small subgroups (14).

Studies assessing the change in the BMD over time in PA patients are scarce. In accordance with previous data, Salcuni et al. showed the same changes in urinary calcium excretion and PTH in 9 of 11 PA patients 6 months after the beginning of treatment (surgery or spironolactone), whereas in 5 of 11 PA patients 1 year after the beginning of treatment, the BMD was significantly increased in the lumbar spine (p < 0.01) (15). Subsequently, Ceccoli et al. also observed a significant improvement in the BMD in the lumbar spine and the Z-scores of the femoral neck and total hip after a mean follow-up of 24 months after treatment of 40 patients with PA, and a significant change in the PTH levels but not in the serum or urinary calcium levels were also observed (16). However, to date, no studies have assessed changes in fracture risk after surgery or spironolactone treatment for PA patients. All of these studies were compared the parameters before-and-after treatment of PA in same patients, and did not compare the results with those of EH patients. So, it is possible to ignore that the reduction in blood pressure itself may have a beneficial effect on bone metabolism.

In summary, bone loss in PA patients may be reversed by MR antagonists or adrenal surgery, but the results are limited by the relatively small sample size. Regarding the changes in serum and urinary calcium levels after treatment, inconsistent results has been observed, which may be due to the significant heterogeneity of studies (inconsistencies in object selection, drug doses, surgical approaches, follow-up time, and laboratory detection methods).

### Serum Aldosterone in Patients With Osteoporosis

The available data show that PA may be a secondary cause of osteoporosis. Given the detrimental effects of excess aldosterone in bone tissue, attention has been focused on whether patients with osteoporosis have a high prevalence of PA and whether bone metabolism influences the renin angiotensin-aldosterone system (RAAS). Two recent studies targeted patients with osteoporosis (OP). Salcuni et al. observed a higher prevalence of PA in 213 OP patients than that in 109 controls (5.2% vs 0.9%) after excluding confounding factors. The prevalence of PA was even higher in patients with the concomitant presence of osteoporosis, hypertension, and hypercalciuria (26.1%) (10). Another recent study by Wu et al. showed that the PAC and ARR were elevated in 186 normotensive postmenopausal women with postmenopausal osteoporosis (PMO) compared with those in 96 osteopenia patients and 42 HS. An increase in false-positive plasma aldosterone/renin ratios (ARRs) was found in the PMO group. PAC was negatively associated with the BMD T-score of the lumbar spine, femur, neck, and total hip (27). Both studies considered the potential interactions between PA and bone from another perspective.

## Discussion

### How Does Excessive Aldosterone Affect Bone Metabolism?

Compared with the association between excess cortisol secretion and bone, less is known about the link between hyperaldosteronism and osteoporosis. However, the detrimental effects of excess aldosterone on bone may be associated with the interaction and co-secretion of aldosterone with cortisol (29–31). In a recent study, Arlt et al. (29) performed a mass spectrometry–based analysis of 24-hour urine steroid in 174 newly diagnosed patients with PA in comparison to that in controls. This suggested that the secretion of cortisol in patients with PA is significantly increased and is only exceeded by the glucocorticoid output of patients with clinically overt adrenal Cushing syndrome. On the one hand, excess cortisol has a direct detrimental effect on bone health, and the underlying mechanisms are beyond the scope of this review and have been reviewed elsewhere (4). On the other hand, excess aldosterone or cortisone activates MR receptors, and subsequent expansion of the intravascular volume results in decreased proximal tubular resorption, thereby increasing the distal delivery of Na+, Mg2+ and Ca2+; however, mineralocorticoids promote distal tubular Na^+^ resorption without increasing Mg^2+^ and Ca^2+^ excretion. Thus, a high urinary calcium level may be present. A similar response was also found in feces; however, only milligram quantities of Ca^2+^ and Mg^2+^ were excreted through this route (32). The decrease in Ca^2+^ and Mg^2+^ led to secondary hyperparathyroidism, with increases in bone resorption to ensure Ca^2+^ homeostasis. Spironolactone treatment attenuated the loss of Ca^2+^ at each site (33).

In addition, several possible mechanisms may explain the direct effects of aldosterone on bone health. First, mineralocorticoid receptors are expressed in human and rat osteoclasts, osteocytes, and osteoblasts (34) as well as in normal and adenomatous parathyroid tissue (24), and treatment with MR antagonists was shown to attenuate bone loss (15, 16), suggesting a direct effect of aldosterone on bone health and the potential bidirectional interaction between aldosterone and PTH. Second, aldosteronism may account for oxidative/nitrosative stress in a rat model by resulting in a proinflammatory phenotype. Oxidative stress and inflammation lead to systemic effects, including the increased excretion of divalent cations, diminished osteogenesis (35), increased osteoblast and osteocyte apoptosis, and reduced bone formation. spironolactone treatment prevented the development of the proinflammatory phenotype (33). Third, a genome‐wide study focused on identifying new candidate genes associated with osteoporosis indicated that genes involved in aldosterone signaling in epithelial cells may be linked to osteoporosis, supporting the potential relationship between aldosterone and bone (36). Moreover, a very recent study immunolocalized Ca metabolism-related receptors (calcium sensitive receptor, vitamin D receptor, and PTH receptor) in normal adrenal glands and aldosterone-producing adenomas, which demonstrated that aldosterone biosynthesis was regulated by calcium (37). Therefore, excess aldosterone biosynthesis may be related to bone resorption.

### Primary Aldosteronism and Hyperparathyroidism

In the present study, subjects with PA had higher serum PTH levels compared with those patients with essential hypertension or normal subjects. So whether the prevalence of hyperparathyroidism in patients with PA was affected? In fact, reports about coincident PA and primary hyperparathyroidism are increasing (38). In a recent study, Asbach et al. reported the prevalence of primary hyperparathyroidism was 1.2% in a retrospective series of 503 patients with PA, and was 2.1% in 141 prospective PA patients, higher than in normal subjects (2.33‰ in women, 0.85‰ in women). They also observed that secondary hyperparathyroidism was common in patients with PA (39, 40). Meanwhile, Concistre et al. reported the prevalence of primary hyperparathyroidism in patients with PA was 2.6% (8/306) (41). As we know, very rarely, PA and hyperparathyroidism can occur together in Multiple Endocrine Neoplasia Type 1 (MEN1), which is inherited in an autosomal dominant manner with the prevalence 2–20/100,000 (42). Nevertheless, it must be noted that MEN1 could not be ruled out in the above two studies. Moreover, few papers focused on the change of aldosterone in primary hyperparathyroidism. In two case reports, the hypertension and secondary aldosteronism were resolved after the surgical treatment of primary hyperparathyroidism (43, 44), and in another study, decreased aldosterone after the extirpation of the parathyroid adenoma was observed in 16 patients (45), which indicated that aldosteronism might be caused by primary hyperparathyroidism occasionally. Above evidences indicated a bidirectional interaction between aldosterone and PTH, but details and concrete mechanisms need more researches to elucidate.

### Strengths and Limitation

To our knowledge, this is the first systematic review aimed to assess the association between PA and osteoporosis. However, some limitations must be pointed out: First, the reliability of the data was limited by the small sample and the observational design of this study. Second, the results included were limited to full-text articles published in English, the problem of incompletely retrieval may exist, and there may be possible bias due to the heterogeneity of the analyzed studies. Third, laboratory testing methods were inconsistent in different studies, which may affect the accuracy of the study. Forth, the time of follow-up was not long enough. Fifth, few data can be acquired with regard to BMD and fractures, so only descriptive analyses had been conducted.

## Conclusion

Excess aldosterone may be associated with increased serum PTH levels, hypercalciuria, which can be reversed by adrenalectomy or spironolactone treatment. BMD evaluated by DXA may not be an ideal index to reflect the bone involvement caused by aldosterone. PA may be a secondary cause of osteoporosis and is associated with an increased risk of bone fracture. The clarification of the relationships between PA and bone metabolism requires additional prospective randomized controlled studies in a large sample.

## Data Availability Statement

All datasets presented in this study are included in the article/supplementary material.

## Author Contributions

SS and TC designed the study and participated in data collection. SS performed the meta-analysis and drafted the manuscript. HT and YR partially conceived the research idea. CL edited the manuscript. All authors contributed to the article and approved the submitted version.

## Funding

This study was supported by a grant from the 1.3.5 project for disciplines of excellence, West China Hospital, Sichuan University (ZYGD18022).

## Conflict of Interest

The authors declare that the research was conducted in the absence of any commercial or financial relationships that could be construed as a potential conflict of interest.
